# Current challenges in treatment options for visceral leishmaniasis in India: a public health perspective

**DOI:** 10.1186/s40249-016-0112-2

**Published:** 2016-03-08

**Authors:** Om Prakash Singh, Bhawana Singh, Jaya Chakravarty, Shyam Sundar

**Affiliations:** Infectious Disease Research Laboratory, Department of Medicine, Institute of Medical Sciences, Banaras Hindu University, Varanasi, India

**Keywords:** Visceral leishmaniasis, Treatment, Drug resistance, Multidrug therapy

## Abstract

Visceral leishmaniasis (VL) is a serious parasitic disease causing considerable mortality and major disability in the Indian subcontinent. It is most neglected tropical disease, particularly in terms of new drug development for the lack of financial returns. An elimination campaign has been running in India since 2005 that aim to reduce the incidence of VL to below 1 per 10,000 people at sub-district level. One of the major components in this endeavor is reducing transmission through early case detection followed by complete treatment. Substantial progress has been made during the recent years in the area of VL treatment, and the VL elimination initiatives have already saved many lives by deploying them effectively in the endemic areas. However, many challenges remain to be overcome including availability of drugs, cost of treatment (drugs and hospitalization), efficacy, adverse effects, and growing parasite resistance. Therefore, better emphasis on implementation research is urgently needed to determine how best to deliver existing interventions with available anti-leishmanial drugs. It is essential that the new treatment options become truly accessible, not simply available in endemic areas so that they may promote healing and save lives. In this review, we highlight the recent advancement and challenges in current treatment options for VL in disease endemic area, and discuss the possible strategies to improve the therapeutic outcome.

**Electronic supplementary material:**

The online version of this article (doi:10.1186/s40249-016-0112-2) contains supplementary material, which is available to authorized users.

## Multilingual abstracts

Please see Additional file 1 for translations of the abstract into the six official working languages of the United Nations.

## Introduction

More than 1 billion people are affected by one or more neglected infectious diseases worldwide [1, 2], for which we lack effective, affordable, and easy to use drugs as well as other control methods. Visceral leishmaniasis (VL), also known as kala-azar, is one of the disorders in this group caused by a protozoan parasite, *L. donovani* and/or *L. infantum*, which is transmitted by the bite of an infected sand fly, *Phlebotomus argentipes* in the Indian subcontinent (ISC) [3]. VL results in prolonged fever, anemia, splenomegaly, wasting; and is fatal when left untreated [4]. There are approximately 200–400 thousands new cases every year occurring predominantly in just six countries: India, Bangladesh, Sudan, South Sudan, Ethiopia and Brazil [5]. More specifically, >10,000 cases occur in India alone every year and the state of Bihar accounts for majority of these cases. These figures, however, do not reflect the true social impact of this disease because VL has a focal distribution which is devastating to the affected communities. The cost of treatment is important when patients need to pay for treatment as ~75 % of the VL cases in Bihar live below the poverty threshold of less than US $ 1.0 a day, and this is similar in other endemic countries although exact data are scarce [6]. Poverty seriously affects the prognosis of VL because most of the patients and their families have to pay for diagnosis, drugs and hospital care, and this is often half or more of the annual household income [7]. As a result, families with a VL infected member descend deeper into poverty.

**Fig. 1:**
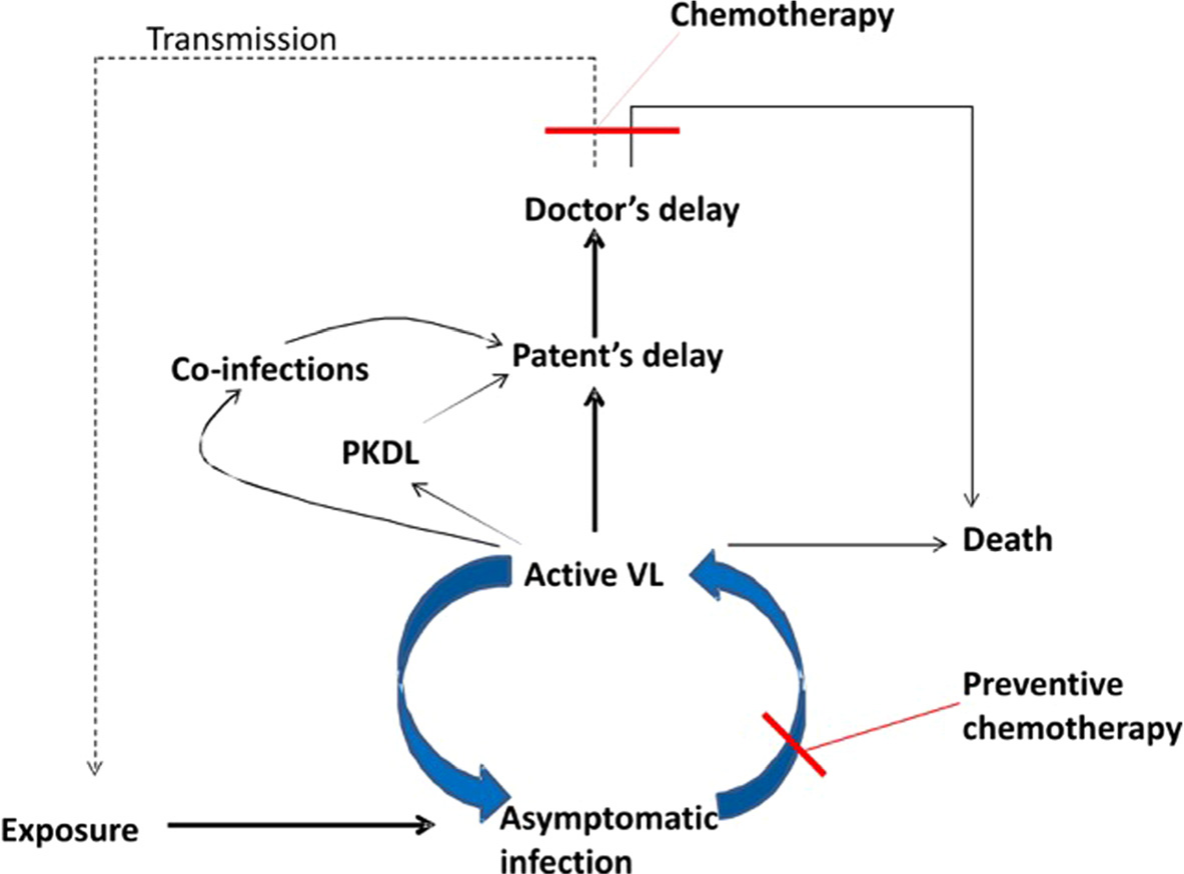
Schematic of intervention model based on VL chemotherapy

VL has never been featured as high priority for drug development programs funded by the pharmaceutical industries because it disproportionately affects the poor people in developing countries and are unlikely to yield good returns on R&D costs. In 2005, the governments of India, Bangladesh and Nepal signed a joint memorandum of understanding to eliminate VL with the aim to reduce the incidence to less than 1 per 10,000 people at sub-district level by the year 2015 [8] which has recently been extended to the year 2017 [9]. Because of the anthroponotic nature of the transmission of *L.donovani* in the ISC, the use of quality drugs is not only a therapeutic tool, but also a tool for VL control. Indeed, human beings are the only known reservoir of *L.donovani*, therefore, identification and proper treatment of parasite carriers will reduce the parasite biomass and prevents onward transmission and deaths (Fig. 1). These factors urge for search of new, effective, less toxic and simplified treatments to replace or complement the few currently available drugs. Unfortunately, no new antileishmanial drugs are expected in near future, because very few drugs are in the R&D pipeline at various stages of development [10]. Furthermore, resistance to first line treatment has long plagued effective treatment of VL in India, making second line treatments and extended hospitalization more common. In India, about 5–10 % of patients with VL, after recovery of acute illness, may develop a chronic cutaneous form called Post kala-azar dermal leishmaniasis (PKDL) that requires prolonged and expensive treatment [11]. The emergence of HIV and its association with VL results in a deadly synergy. A significant number of patients in Bihar are living with HIV-VL co-infection [12], and we expect to see more HIV-VL co-infections in near future which will further generate major therapeutic challenges [13]. In the following sections, we have briefly reviewed the current treatment of VL in endemic areas of Bihar, India, and discuss the challenges and its possible solutions.

## Treatment options for VL

There is no vaccine available for VL; hence control of VL exclusively depends on chemotherapy. Available treatment options for VL are limited and not up to satisfactory standards due to problem relating to efficacy, adverse effects, increasing drug resistance, high cost and need for hospitalization to complete the full dose of treatment. So far, many clinical trials have been done in India to optimize the therapeutic regimens and to protect the efficacy of limited number of available anti-leishmanials (Tables 1 and 2). A full list of completed, ongoing and proposed clinical trials for leishmaniasis in various parts of the world is available on clinical trial registry site (https://clinicaltrials.gov). Indeed, important recent advances have been made in the area of VL treatment that if implemented effectively could eliminate this disease from most endemic parts of the world. Table 3 lists the rank wise recommended dose of anti-leishmanial drugs for VL treatment.

**Table 1:** Currently available anti-leishmanial drugs for treatment of VL: product name, cure rate, mode of action on parasite, advantages and limitations

S.N.	Drugs	Marketing authotization and commercial product	Cure rate	Mechanism of action	Advantages	Limitations
1	Pentavalent antimonials	Albert David, India (generic SSG); Wellcome (Pentostam); Sanofi Aventis (Glucantime)	39–95 % depending on geographical condition (50 % in Bihar)	Act as prodrug, inhibit trypanothione reductase, increase the ROS	Low cost and easy available	Parasite resistance especially in India, cardiotoxic, 30 day iv/im treatment in hospital, painful injections
				Inhibit macromolecular biosynthesis in amastigotes		
2	Amphotericin B	Life care, India	>98 % in all regions	Form complexes with sterols mainly ergosterols of parasite membrane leading to increase permeability resulting in cell death	High efficacy, 1st line treatment in case of antimonial resistance	Dose-limiting renal toxicity, 15–30 day slow iv infusion treatment over 4–6h in hospital, hypokalaemia
		(Fungitericin); Bristol –Myers (Fungisone)				
3	Liposomal amphotericin B	Gilead (AmBisome);	>98 %	Targeted delivery of drug to infected macrophages and kill the parasites as AmB	Highest therapeutic index of available VL drugs, No need of hospitalization, substantially less nephro-toxic than AmB	Expensive, requirement of cool chain temperature maintainance
		Lifecare Innovation (Fungisome); Bharat Serum and Vaccine (Amphomul);				
		Sequus Pharmaceuticals (Amphocil);				
		ENZON Pharmaceuticals (Abelcet);				
		Lifecare Innovation (Kalsome)				
4	Miltefosine	Paladin Labs (Impavido)	94–97 %	Modulate cell surface receptors and inositol metabolism of parasites, and cell death is mediated by apoptosis, Inhibit the cytochrome C oxidase	Oral drug, Safe in HIV-VL co-infection	Teratogenic in experimental animals, originally developed as an anti- cancer drug, expensive, GI toxicity, hepato- & renaltoxicity in phase-4
5	Paromomycin	Gland Pharma/iOWH	94 % (India)	Binds to 30S ribosomal subunit and interfere with protein biosynthesis, decrease the membrane potential of parasite	Cheapest drug	An aminoglycoside, therefore nephro- and ototoxicity possible, but have not been reported in VL patients, although reversible high tone audiometric shift may occasionally occur during treatment
6	Pentamidine	Sanofi Aventis (Pentacarinat)	70–80 %	Inhibit mitochondrial topoisomerase II and inhibit the transcription process	Effective in combination therapy	Gastointestinal adverse effect, hypotension, diabetes mellitus

**Table 2:** Summary of studies and clinical trials on monotherapy in treatment of visceral leishmaniasis in India

Authors	Year	Drug	Type of study	Patients (N)	Treatment Scheme	Cure rate	Reference
Thakur et al.	1988	*Pentavalent antimonials*	Randomized dose finding trial of SSG	371	20 vs. 10 mg Sbv+/kg/day for 28 days (i.m)	60–100 %	[108]
Thakur et al.	1991	*Pentavalent antimonials*	Randomized trial	312	20 mg Sbv+/kg/day for 20, 30 & 40 days (i.m)	71–94 %	[109]
Mishra et al.	1991	Amphotericin B deoxycholate	Non-Comparative study	15	0.5 mg/kg/day for 28 days (i.v)	93 %	[110]
Mishra et al.	1992	Amphotericin B deoxycholate vs. Pentamidicine	Randomized comparative study in antimony unresponsive patients	120	AB: 0.5 mg/kg/on alternate day for 14 days (i.v)	77–98 %	[111]
					Pentamidinine: 4 mg/kg on alternate days for 20 injections (i.m)		
Thakur et al.	1993	Amphotericin B deoxycholate	Non comparative study on SSG resistant patients	300	1.0 mg/kg on alternate day, total dose 20 mg/kg (i.v)	99 %	[19]
Thakur et al.	1993	Amphotericin B deoxycholate vs. *Pentavalent antimonials*	Randomized controlled comparative trial	150	AB: 1.0 mg/kg on alternate day, total dose 20 mg/kg (i.v)	80–100 %	[112]
					SSG: 20 vs. 10 mg Sbv+/kg/day for 30 days (i.m)		
Mishra et al.	1994	Amphotericin B deoxycholate vs. *Pentavalent antimonials*	Randomized controlled comparative trial	80	AB: 0.5 mg/kg on alternate day for 14 days (i.v)	62–100 %	[113]
					SSG: 20 mg Sbv+/kg/day for 40 days (i.m)		
Thakur et. al.	1994	Amphotericin B deoxycholate	Radomized dose finding study	80	1.0 mg/kg on daily vs. alternate day, total dose 20 mg/kg (i.v)	100 %	[114]
Thakur et. al.	1994	Amphotericin B deoxycholate	Dose finding study	120	1.0 mg/kg/day on incremental increasing dose vs. contantdoase, total dose 20 mg/kg (i.v)	100 %	[115]
Giri	1994	Amphotericin B deoxycholate	Non comparative study in pentamidinde relapse patients	25	0.75 mg/kg on alternate days (i.v) total 15 infusions	100 %	[116]
Giri& Singh	1994	Amphotericin B deoxycholate	Non comparative study in antimony relapse patients	100	0.75 mg/kg on alternate days (i.v) total 15 infusions	100 %	[117]
Jha et. al.	1995	Amphotericin B deoxycholate	Dose finding study in multidrug resistant patients	34	1.0 mg/kg/day on alternate days (i.v) total 10–15 infusions		[18]
Thakur et.al.	1996	Amphotericin B deoxycholate	Randomized dose finding study	288	1.0 mg vs. 0.75 mg vs. 0.5 mg/kg/day for 20 days (i.v)	79–99 %	[118]
Thakur et.al.	1998	Amphotericin B deoxycholate	Randomized dose finding study	130	1.0 mg/kg/day for 20 days (i.v) vs. escalating dose for 5 days then 1.0 mg/kg/day for 43 days	99 %	[119]
Thakur et.al.	1999	Amphotericin B deoxycholate	Non comparative dose finding study	938	1.0 mg/kg/day for 20 days (i.v)	99.2 %	[120]
Thakur & Ahmed	2001	Amphotericin B deoxycholate	Non comparative study	309	1.0 mg/kg/day for 20 days (i.v)	95.8 %	[22]
Thakur & Narayan	2004	Amphotericin B deoxycholate vs. SSG	Randomized comparative study	60	AB: 1.0 mg/kg/day for 20 days (i.v)	46.6 and 100 %	[121]
					SSG: 20 vs. 10 mg Sbv+/kg/day for 28 days (i.m)		
Singh et.al.	2010	Amphotericin B deoxycholate	Randomized study in children’s	605	1.0 mg/kg/day for 15 days daily vs. alternate days (i.v)	100 %	[122]
Thakur et.al.	1996	Liposomal Amphotericin B (LAB)	Randomized open study of different schedule	30	2 mg/kg/day on day 1, 2, 3, 4, 5, 6 and 10 vs. days 1, 2, 3, 4 and 10	100 %	[123]
Thakur et.al.	2001	Liposomal Amphotericin B vs. Amphotericin B deoxycholate	Randomized trial	34	LAB: 15 mg/kg single dose (i.v)	100 %	[28]
					AB: 1.0 mg/kg/day for 20 days (i.v)		
Sundar et.al.	2001	Liposomal Amphotericin B	Open label dose finding study	91	5 mg/kg (single dose) vs. 1 mg/kg for 5 days (iv)	91 and 93 %	[30]
Sundar et.al.	2002	Liposomal Amphotericin B	Randomized double-blind dose ranging multicentre trial	84	0.75 mg/kg/days for 5 days (i.v)	89, 93 and 96 %	[124]
					Vs		
					1.5 mg/kg/days for 5 days (i.v)		
					Vs		
					3.0 mg/kg/days for 5 days (i.v)		
Sundar et.al.	2003	Liposomal Amphotericin B	Open label non comparative study	203	5.0 mg/kg/days for 5 days (i.v)	90 %	[29]
Sundar et.al.	2004	Liposomal Amphotericin B vs. Amphotericin B deoxycholate	Randomized comparative study	153	AB: 1.0 mg/kg/day for 15 dose on alternate days (i.v); LAB: 2.0 mg/kg/day for 5 days (i.v) Vs. Amphotericin B lipid complex 2 mg/kg/day for 5 days (i.v)	96, 96 and 92 %	[125]
Sinha et.al.	2010	Liposomal Amphotericin B	Observational cohort study	251	5.0 mg/kg/day (i.v) on 0,1,4, and 9	98.8 %	[126]
Sundar et.al.	2010	Liposomal Amphotericin B vs. Amphotericin B deoxycholate	Open label randomized controlled non inferiority study	412	LAB: 10.0 mg/kg/day (i.v) single dose	95.7 and 96.3 %	[27]
					AB: 1.0 mg/kg/day for 15 alternate dose (i.v)		
Thakur et.al.	1984	Pentamidine	Non-comparative study in SSG unresponsive patients	86	4 mg/kg/(i.m) for 15 dose (total dose 60 mg/kg)	93.4 %	[127]
Thakur et.al.	1991	Pentamidine	Randomized controlled comparative study	312	Group1: 4 mg/kg (i.v) 3 times weekly	78, 84 and 98 % respectively	[128]
					Group2: 4 mg/kg (i.v) 3 times weekly + SSG 20 mgSbv+/kg (i.m) daily for 20 days		
					Group3: 4 mg/kg (i.v) 3 times weekly until spleen were free from parasite + SSG 20 mgSbv+/kg (i.m) daily for 20 days		
Mishra et.al.	1992	Pentamidine	Randomized controlled comparative study	120	Pentamidine: 4 mg/kg (i.m) on alternate days (total 20 dose)	77 and 98 %	[111]
					AB: 0.5 mg.kg (i.v) on alternate days		
Das et.al.	2001	Pentamidine	Randomized controlled comparative study	158	Group1 : 2 mg/kg/day (i.m) on alternate days + oral allopurinol 15 mg/kg/day in 3 divided dose for 30 days	91.2 and 74.3 %	[67]
					Group 2 : 4 mg/kg/day (i.m) on alternate days for 30 days		
Das et.al.	2009	Pentamidine	Randomized controlled comparative study	82	Group 1: AB- 1 mg/kg/day alternate days for 15 days (i.v)	92 and 73 %	[129]
					Group 2: Pentamidine- 4 mg/kg/day alternate days (i.m)		
Jha et.al.	1998	Paromomycin	Randomized controlled comparative study	120	Group1: 12 mg/kg/day for 21 days (i.m)	76.7, 96.7, 96.7, and 63.3 %	[130]
					Group 2: 16 mg/kg/day for 21 days (i.m)		
					Group 3: 20 mg/kg/day for 21 days (i.m)		
					Group 4: SSG 20 mg Sbv+/kg/day for 30 days (i.m)		
Sunder et.al.	2007	Paromomycin	Randomized controlled phase III open label comparative study	667	Group 1 : Parmomycin 11 mg/kg/day for 21 days (i.m)	94.6 and 98.8 %	[49]
					Group 2 : AB 1 mg/kg/day for 30 days (i.v)		
Sunder et.al.	2009	Paromomycin	Randomized open label study	329	Group 1 : 11 mg/kg/day for 14 days (i.m)	82–92.8 %	[131]
					Group 2 : 11 mg/kg/day for 21 days (i.v)		
Sinha et.al.	2011	Paromomycin	Phase IV open label study	506	11 mg/kg/day for 21 days (i.m)	94.2 %	[132]
Sundar et al.	1998	Miltefosine	Phase-I/II safety and efficacy trial	30	50 mg-250 mg/day for 28 days (oral)	20–100 %	[133]
Jha et al.	1999	Miltefosine	Phase II randomized open label, dose finding	120	50 mg/day for 6 weeks; 50 mg/day for 1 week followed by 150 mg/day for 3 week; 100 mg/day for 4 week; 100 mg/day for 1 week followed by 150 mg/day for 3 week	93–97 %	[134]
Sundar et al.	1999	Miltefosine	Phase II comparative clinical trial	45	100-200 mg/day for 28 days	94–100 %	[135]
Sundar et al.	2000	Miltefosine	Phase II, Comparative study	54	100 mg/day for 14 days, 21 days or 28 days	88–100 %	[136]
Sundar et al.	2002	Miltefosine	Randomized open label comparative study	398	Miltefosine: 50-100 mg/day for 28 days	97–100 %	[58]
					AmB:1 mg/kg/day (i.v) for 15 days		
Sundar et al.	2003	Miltefosine	Open label phase II dose ranging study in childrens	39	1.5 or 2.5 mg/kg/day for 28 days	88–90 %	[137]
Bhataacharya et al.	2004	Miletfosine	Phase II trial in childrens	80	2.5 mg/kg/day for 28 days	94 %	[138]
Singh et al.	2006	Miltefosine	Prospective multicentric cross sectional study	125	Miltefosine: 2.5-100 mg/kg/day for 28 days	91.3–93.2 %	[139]
					AmB: 1 mg/kg/day (i.v) for 15 days		
Bhattacharya et al.	2007	Miltefosine	Phase IV open label single arm trial	2109	2.5–100 mg/kg/day for 28 days	93.6–96.6 %	[140]
Sundar et al.	2012	Miltefosine	Open label comparative study	567	50–100 mg/kg/day for 28 days	90.3 %	[61]

**Table 3:** Recommended treatment regimens for VL in Indian subcontinent (ranked by preferences)

Anthroponotic VL caused by *L.donovani* in India, Bangladesh, Bhutan and Nepal	
1.	Liposomal amphotericin B: 3–5 mg/kg per daily dose by infusion given over 3–5-day period up to a total dose of 15 mg/kg (A) by infusion or 10 mg/kg as a single dose by infusion (A).
2.	Combinations (co-administered) (A)
	• liposomal amphotericin B (5 mg/kg by infusion, single dose) plus miltefosine (daily for 7 days, as below) • liposomal amphotericin B (5 mg/kg by infusion, single dose) plus paromomycin (daily for 10 days, as below) • miltefosine plus paromomycin, both daily for 10 days, as below.
3.	Amphotericin B deoxycholate: 0.75–1.0 mg/kg per day by infusion, daily or on alternate days for 15–20 doses (A).
4.	Miltefosine: for children aged 2–11 years, 2.5 mg/kg per day; for people aged ≥12 years and <25 kg body weight, 50 mg/day; 25–50 kg body weight, 100 mg/day; >50 kg body weight, 150 mg/day; orally for 28 days (A) or Paromomycin: 15 mg (11 mg base) per kg body weight per day intramuscularly for 21 days (A).
5.	Pentavalentantimonials: 20 mg Sb5+/kg per day intramuscularly or intravenously for 30 days in areas where they remain effective: Bangladesh, Nepal and the Indian states of Jharkhand, West Bengal and Uttar Pradesh (A).

Note: Amphotericin-B or Liposomal amphotericin B at higher dose should be used as rescue treatment in case of non-response

Source: WHO Technical Report Series (2010) Control of the leishmaniasis. WHO,Geneva [83]

Grade of evidence (A)- evidence based on at least one randomized controlled trial

### Pentavalent Antimonials

Past experiences have confirmed that response to a drug varies from region to region. For example, in hyper-endemic regions of India and adjoining areas of Nepal, pentavalent antimonials (Sb^v^ also known as sodium stibogluconate) has lost its efficacy with the result that about two thirds of patients in some of these areas are refractory to Sb^v^ treatment [14]. However, in Bangladesh, the situation is different as no resistance to Sb^v^ has been officially reported. Sb^V^was recommended and used in Bangladesh till 2009 as a first line drug [15]. Moreover, Sb^v^ are still the first-line drugs in many countries worldwide for all clinical forms. Major side effects are cardiac arrhythmias, prolonged QT interval, ventricular premature beats, ventricular tachycardia and ventricular fibrillation [16]. There are accumulating evidence suggesting that Sb^v^ has a dual mode of action, and acts on both the parasite and the infected macrophage. Upon contact with infected macrophages, Sb^v^ stimulates the macrophages to kill the intracellular parasites and when reaching the parasite, Sb^v^ is reduced to Sb^III^, which can directly kill the parasite inside phagolysosome by inhibiting trypanothoine reductase (an enzyme that recycle oxidized trypanothione to keep the trypanothione in reducing state) [17]. Pentamidine, a diamidine compound, was the first drug to be used in Sb^v^ refractory patients, and cured most patients initially, but after a decade its efficacy also declined from ~100 to 70 % in hyper-endemic areas of India.

### Amphotericin B Dexoycholate (AmB)

Amphotericin B (AmB) was initially recommended in India for treatment of patient’s refractory to Sb^v^ [18, 19]. However, due to increasing unresponsiveness of Sb^v^ in endemic areas, it is currently being used as first line drug for VL treatment. AmB formulated with sodium deoxycholate was the first parenteral amphotericin B preparation available commercially as Fungizone (Bristol-Meyer-Squibb). Several clinical trials have been conducted till date for treatment of VL involving AmB (reviewed in ref.[20, 21]) with excellent cure rate (~100 %) at dose of 0.75–1.0 mg/kg for 15–20 intravenous infusions [22, 23]. The drug has high safety and efficacy; however, prolonged hospitalization, adverse reactions like high fever with rigor and chills, and the need to close monitoring of renal functions and electrolyte levels are well-recognized drawbacks of AmB treatment (Table 2 enlists the studies in India with AmB). The mechanism of action of AmB is still not fully investigated but it is assumed that it interferes with the ergosterol in the cell membrane of *Leishmania* parasite, causing changes in the membrane permeability and leakage of intracellular components [24]. Cell death occurs in part because of these permeability changes, but other mechanisms may also contribute to AmB antifungal activity. AmB is not active *in vitro* against organisms that do not contain sterols in their cell membranes (e.g., bacteria). Binding to sterols in mammalian cells (e.g., certain kidney cells, erythrocytes) may be responsible for the toxicities associated with AmB (reviewed in ref. [25]).

### Lipid formulations of Amphotericin B (L-AmB)

The advent of Liposome Technology in mid 1960s and subsequently its application for minimizing dose-limiting toxicity has added a new paradigm in AmB treatment, providing a highly effective and safe therapy for many forms of systemic mycosis. There are six lipid formulations of amphotericin B available commercially that differ from each other with respect to dose, efficacy and toxicities. These are: i) liposomal amphotericin B (AmBisome®; Gilead Sciences); ii) Amphotericin B lipid complex (Abelcet®; ENZON Pharmaceuticals Inc.); iii) AmB cholesteryl sulfate complex, also called AmB colloidal dispersion [ABLC] (Amphocil; Sequus Pharmaceuticals); iv) FUNGISOME™ (Lifecare Innovation Pvt Ltd); v) AmB emulsion (Amphomul, Bharat Serum and Vaccines, India); and vi) amphiphilic L-AmB (KALSOME™10, Life care Innovation, Pvt. Ltd, India). Among these, Ambisome® is tested in most of the clinical trials and is probably the most efficacious of all anti-leishmanial drugs currently available [26]. Most of the clinical trials of L-AmB for the treatment of VL have taken place in India, where more than 10 different regimens have been tested (Table 2). Most have been open-label, dose-finding studies or randomized controlled comparisons. Indian experience has demonstrated that L-AmB caused substantially less toxicity than conventional AmB or amphotericin B lipid complex (ABLC), but high cost is the major drawback. Much of research has been focused to reduce the course of L-AmB whilst retaining its efficacy, to limit the cost to patients. Sundar et al. showed that 15 mg/kg of Ambisome® (3 mg/kg on each of 5 injections) cured 96 % patients [27]. Later on in separate study by Thakur *et al*. [28], and Sundar *et al*. [27, 29, 30] have demonstrated the efficacy and safety of Ambisome® achieving efficacy rates in excess of 90 % in single doses of 5–15 mg/kg (Table 2), making it an excellent treatment option for VL in the ISC. Low toxicity of L-AmB has made it best treatment option for HIV-VL co-infection patients. In a study by Sinha *et al*., excellent long term survival and retention rate were obtained; however, relapse within 2 year remained frequent [31]. It can be given safely in doses as high as 30–40 mg/kg body weight in HIV-positive VL patients [32, 33]. It has been speculated that lipid formulations enhance uptake by macrophages (the site of parasite replication) which results in the localization of the drug in the macrophage abundant areas in the body. Ambisome® is currently being used as a first line drug for the treatment of VL in India, under the kala-azar elimination program.

Among other lipid formulations, Fungisome™ with the dose 7.5 mg/kg daily for 2 days showed 100 % cure rate (without any serious adverse effect) in an open label randomized study [34, 35]. Abelcet has shown cure rate 90–100 % at total dose of 10–15 mg/kg in Sb^V^ resistant patients [36]. Amphocil was evaluated at three different doses (7.0, 10 and 15 mg/kg) which showed final cure rate up to 97 % [37]. Amphomul (single dose: 15 mg/kg body weight) was found highly effective and safe for treatment of VL [38]. KALSOME^TM^ is still not tested on human VL, however, in murine model with 7.5 mg/kg double dose results in almost complete clearance of parasites from both liver and spleen [39].

### Paromomycin

Paromomycin is a broad-spectrum aminoglycoside antibiotic produced from culture filtrates of *Streptomyces krestomyceticus* and with activity against a variety of Gram-positive and negative organisms, mycobacteria, protozoa. The anti-leishmanial activity of paromomycin was first demonstrated in the 1960s [40, 41] and subsequently confirmed *in vitro* and *in vivo* [42]. This drug was first tested in Kenya in 1980s for treatment of human VL [43]. It was registered for treatment of patients with VL in India in 2006 by Gland Pharma Ltd., Hyderabad, India, who is now the sole manufacturer for intramuscular paromomycin worldwide [44]. Several clinical trials have been conducted in Kenya, Sudan and India [43, 45–48], and all these studies have reported that paromomycin, when used as a single agent or in combination with sodium stibogluconate was highly efficacious and well tolerated in the treatment of VL. High efficacy rates for paromomycin (i.m) injection (dose-11 mg/kg for 21 days) has been reported to 98.4 % with initial cure (defined as the initial response after complete treatment), and the final cure (defined as a complete response with no evidence of relapse up to 6 months after an initial cure of 21 days of treatment) was approximately 94.6 % [49]. Shortening the course of this drug from 21 to 14 days has subsequently shown inferior cure rate [50]. Pain at injection site, elevated liver function tests (LFTs); fever, proteinuria, vomiting, elevations in alkaline phosphatase and bilirubin values are the main adverse events associated with this drug. The mechanism of action of paromomycin is thought to be interference with protein synthesis in the ribosome of the target organism and inhibit the respiration [51].

### Miltefosine

Miltefosine is an alkyl phospholipid compound was the first effective oral anti-leishmanial agent in VL, and registered for the treatment of VL in India in 2002, Germany in 2004, Colombia in 2005 and Bangladesh in 2006 (reviewed in ref. [52]). Miltefosine was originally intended for breast cancer and other solid tumours [53]. However, due to dose limiting gastro-intestinal toxicity, it could not be developed as an oral agent in cancer [54]. Evidence of excellent anti-leishmanial activity both *in-vitro* and in experimental animal models [55–57] prompted the clinical assessment of oral miltefosine in human VL in1996 [53]. Miltefosine was licensed for use in VL patients in India in 2002 following a Phase III clinical trial in which 94 % long term cure rate was observed in a dose 50–100 mg/day for 28 days [58]. It was licensed in Europe for treatment of HIV-VL co-infected patients in 2005 [59, 60]. However, because of its teratogenic effect in animals and its long-term residual persistence shown in humans, there is some concern on unrestricted use of the drug in women of child-bearing-age. Being orally administrable, miltefosine has a big advantage of domiciliary treatment. However, it has the drawback of poor compliance due to its prolonged treatment regimen well beyond the period in which there is almost a complete physical recovery of these patients. Also, with a long half-life of seven days, the chances of parasites developing resistance are high. A recent studies showed that after a decade of this drug use in Bihar (India), 6.8 % of patients relapsed within 6 month of treatment [61]. These considerations suggest for alternative strategies to protect this drug from failure due to non-compliance or resistance and to prolong its clinically useful life. One such way is to combine a short course of miltefosine, to which compliance should be high, with a short course of another effective anti-leishmanial compound to obtain complete cure and to protect against single-agent resistance (Table 2).

### Sitamaquine

Sitamaquine is another oral drug after miltefosine developed by Walter Reed Army Institute of Research (WRAIR, USA) in collaboration with GlaxoSmithKline (UK). Clinical trials using this drug have been completed in India, Kenya and Brazil [62–65] with cure rate ranging from 27 to 87 %. A major side effect was nephrotoxicity. Exact mechanism of this drug is not known but it is thought that it targets succinate dehydrogenate causing oxidative stress in *leishmania* parasites [66]. Further development of this molecule has been abandoned.

### Pentamidine

Pentamidine, an aromatic diamidine that emerged earlier in Bihar, India as a second line drug to circumvent the problems of Sb^V^ resistant in VL patients. However, due to inferior cure rate to AmB and toxicity issues (cardiac, hypotension, diabetes mellitus, gastrointestinal), use of this drug as monotherapy has been abandoned in endemic areas [67]. It is commercially available as Pentacarinat® (Sanofi-Aventis). Pentamidine is currently recommended as secondary prophylaxis in HIV-VL co-infection. The mechanism of action of pentamidine in *Leishmania* and other kinetoplastids is the inhibition of active transport system and DNA-mitochondrial complex [68].

## Multidrug therapy

In VL, multidrug therapy has been advocated for several reasons: i) reduce the treatment time and cost; ii) slow the emergence of parasite resistance as mode of action of drugs will be different; iii) increase the efficacy rate even in the case of co-infection [20]. This strategy of multidrug treatment has been successfully used in the treatment of tuberculosis, malaria and leprosy. It also holds promises especially in complicated situations like HIV co-infection. Ideally, drugs used in combination therapy must be of synergistic and additive effect. One of the best approaches is to use one very active drug with a short half-life with second slow acting drug having a longer half-life to clear the remaining parasites. A comparative overview of different combination therapy studies for treatment of VL in India has been presented in Table 4, which suggests that multidrug therapies are safe and effective.

**Table 4:** Studies on combination therapy for VL in India

Authors	Year	Drug	Type of study	Patients (N)	Treatment scheme	Definite cure (95 % CI)	Reference
Thakur et al.	1991	SSG and Pentamidine	Randomised controlled comparative trial	312	Group-1 : Pentamidine (i.v) 4 mg/kg/day three times weekly until parasitological cure was achieved	Group 1:78 % Group 2: 84 % Group 3: 98 %	[128]
					Group-2: Pentamidine (i.v) 4 mg/kg/day three times weekly + SSG (i.m) 20 mg/kg/day for 20 days		
					Group-3: Pentamidine (i.v) 4 mg/kg/day three times weekly until parasitological cure was achieved + SSG (i.m) 20 mg/kg/day for 20 days		
Thakur et al.	1992	Paramomycin and SSG	Non comparative study	22	Paramomycin (i.v) 12 mg/kg/day +	81.8 %	[47]
					SSG (i.m) 20 mg/kg/day for 20 days		
Thakur et al.	2000	SSG and Paramomycin	Randomized comparative study	149	Group 1: Paramomycin 12 mg/kg/day + SSG (i.m) 20 mg/kg/day for 21 days	Group 1:92.3 % Group 2: 93.8 %, Group 3: 53.1 %	[141]
					Group 2: Paramomycin 18 mg/kg/day + SSG (i.m) 20 mg/kg/day for 20 days		
					Group 3: SSG (i.m) 20 mg/kg/day for 21 days		
Das et al.	2001	Pentamidine and Allopurinol	Randomized controlled comparative trial	158	Group 1: Pentamidine (i.m) 2 mg/kg/day on alternate days + allopurinol (oral) 15 mg/kg/day for 30 days	Group 1: 91.2 %, Group 2: 74.3 %	[67]
					Group 2: : Pentamidine (i.m) 2 mg/kg/day on alternate days for 30 days		
Sundar et al.	2008	L-AmB and Miltefosine	Randomized non-comparative, group sequential	226	Group1: L-AmB (i.v) 5 mg/kg single dose	Group 1: 91 % (78–97);	[142]
					Group 2: L-AmB (i.v) 5 mg/kg single dose + miltefosine 100 mg/day for 10 days	Group 2: 98 % (87–100);	
					Group3: L- AmB (i.v) 5 mg/kg single dose + miltefosine 100 mg/day for 14 days	Group 3: 96 % (84–99);	
					Group 4: L-AmB (i.v) 3.75 mg/kg single dose + miltefosine 100 mg/day for 14 days	Group 4: 96 % (84–99);	
					Group 5: L-AmB (i.v) 5 mg/kg single dose + miltefosine 100 mg/day for 7 days	Group 5: 98 % (87–100)	
Sundar et al.	2010	L-AmB, Miltefosine	Non-randomized multicentric trial	135	L-AmB (i.v) 5 mg/kg for single dose + miltefosine (oral) 2.5 mg/kg/day for 14 days	91.9 %	[143]
Sundar et al.	2011	AmB, L-AmB, Paramomycin, Miltefosine	Open label non-inferiority randomized control trial	634	Group 1: AmB (i.v) 1 mg/kg on alternate days for 30 days	Group 1: 93 % (88–96);	[144]
					Group 2: L-AmB (i.v) 5 mg/kg for single dose + miltefosine (oral) 50 mg/kg for 7 days	Group 2: 98 % (93–99)	
					Group 3: Paramomycin (i.m) 11 mg/kg/day for 10 days	Group 3: 98 % (93–99);	
					Group 4: Miltefosine (oral) 50 mg/day for 10 days + paramomycin (i.m) 11 mg/kg/day for 10 days	Group 4: 99 % (95–100);	

## Immune responses and immunomodulatory activity of anti-leishmanial drugs

One of the major hurdles for developing an effective vaccine for VL, as well as safer and more appropriate drugs and therapies, has been a limited understanding of the precise immune mechanisms required for controlling parasite growth (reviewed in ref. [69]). It has been thought that clinical efficacy of the disease treatment not only depends on direct effect of drugs alone, but an effective immune response also play critical role in final cure (Table 1). The use of biological molecules or compounds to stimulate or modulate innate and cell mediated immunity in order to achieve the therapeutic goal has been tested in both preclinical and clinical studies in treatment of leishmaniasis (reviewed in ref.[69]). For example, Sb^V^ was not able to clear the parasites in T cell deficient BALB/c mice [70] as well as IFNγ and IL-12 gene knockout mice [71, 72]. Treatment with exogenous IL-12 along with Sb^V^ resulted in rapid clearance of *L.donovani* parasites (Table 1). Later on in subsequent separate study, it was reported that treatment with a single-dose anti-IL-10 receptor monoclonal antibody and daily low doses of Sb^v^ dramatically enhance the therapeutic effects of Sb^v^ in experimental mice model [73]. These findings strongly supported the idea that immune mechanisms play an important role in clinical outcome of disease. This was further demonstrated by studies on human VL, where stronger parasitological and clinical cure have been observed with recombinant human IFNγ along with SSG compared with the SSG drug alone from India, Brazil and Kenya [74–76]. Basu *et al.* reported that SAG induces generation of reactive oxygen species (ROS) and nitric oxide (NO) dependent parasite killing via phosphorylation of ERK1/2 and p38 MAPK [77]. Similar immunomodulatory activity is also reported with miltefosine which induces IFN-γ, TNF-α and IL-12 production from macrophages [78]. In PKDL, miltefosine induces significant increase in levels of pro-inflammatory cytokines with concomitant decrease of anti-inflammatory cytokines via up-regulation of activation markers CD16 and CD 86 and down regulation of CD14 in circulating monocytes [79]. AmB has been shown to induce the production of TNF-α [80], IL-6 [81, 82], IL1ß [83–85] and M-CSF [86] from macrophage and monocytes and simultaneous suppression of mRNA expression of IL-10 and TNF-ß [87]. Therefore, immune based therapies in combination with chemotherapy that enhance immune responses to fight VL are of significant clinical interest. Such novel approaches may be of very useful for therapies to stimulate the immune system where patients are immune-compromised, such as those with HIV-VL co-infection.

## Treatment failure and parasite drug resistance

Treatment of VL cases is complicated by patients’ late presentation at an advanced stage of their illness; and treatment outcome mainly depends on the interaction between the drug, the parasite and the human host [88]. Treatment failure is well documented for Sb^v^ but the mechanism is far from being completely understood. Most alarming reports came from Bihar (India), where 65 % of VL-patients were found to be unresponsive to Sb^v^ treatment, while in Nepal, recent reports indicate an unresponsiveness rate of up to 24 % in one district [89, 90]. Recently, it was reported that multiple Sb^v^ resistance mechanism are circulating in the Indian subcontinent [91] including the loss of metal reduction, over-expression of thiol metabolism enzymes, multi drug resistant transporter and reduced drug uptake due to decreased expression of aquaporins in different experimental models [92]. However, knowledge gained from transcriptional profiling studies and proteomic approaches emphasized the involvement of HSP’s, histones, calpain-related proteins and MAPK [93]. While the metabolomics studies have identified many changes and variation in the lipid composition that alters the membrane fluidity [94] and amino acid composition [95]. These facts suggested for the adaptability of the parasite, and therefore, genome plasticity in *Leishmania* which has further been validated by Downing and colleagues, to prove the existence of different genetic background in drug resistant parasites within a single geographical area [96] thereby consolidating the idea for the existence of drug resistant phenotypes in the population. Many other reports for the drug resistance have been available attributing to the role of efflux transporters, aquaporins, and alterations in the intracellular thiol levels in drug resistant clinical isolates has been discussed ahead.

AmB resistance, though rare, has been known to result in changes in the sterol profile where the ergosterol is replaced by its precursor cholesta-5,7,24-trien-3-ol in the membrane of parasite thereby reducing its affinity towards the drug. Amplification in the extra chromosomal DNA which has been directly associated with the resistant phenotypes [97]. The mechanism of resistance has further been explored by the proteomic analysis that shed light on the involvement of energetic pathways which are up-regulated including the glycolytic and TCA cycle while documenting the role of reactive oxygen species (ROS) scavenging pathways and heat shock proteins as additional weapon for protection against the drug induced stress [98]. As mentioned above, the role for the efflux pump (MDR) remains to be the important factor driving the drug resistance together with the thiol machinery for better coping up the ROS induced oxidative stress [99].

Emerging resistance against miltefosine is a matter of serious concern as it is the only available oral anti-leishmanial drug. Incomplete treatment and long half-life of this drug in the circulation has been thought to be one of the factors for driving the parasite machinery for adaptability against the drug induced stress. The exact mechanism of which could be imputed to the allele specific mutations in P-glycoprotein-LdRos3 and LdMT [100] which are responsible for drug uptake in ideal conditions, but the mutation causes the gene inactivation and consequently decreasing the drug accumulation and drug translocation in parasite. Apart from above mentioned reasons increased drug efflux has been another threatening cause of drug resistance. Mode of parasite killing involves the induction of apoptosis by accumulation of ROS but the resistant phenotype has been known to alter the cellular machinery and thereby reducing ROS mediated apoptotic phenomenon. The resistant phenotypes have also been known to be armed for coping up the oxidative stress by up-regulating several important enzymes as superoxide dismutase and ascorbate peroxidase [101].

Paromomycin, another drug targeting the parasite mitochondrial protein synthesis machinery has led the parasite to emerge as a strong survivor with reduced drug binding to the surface, increased translational activities with up-regulation of glycolytic enzymes and intracellular proteins, high expression levels of ATP-Binding Cassette (ABC) transporters [51]. The developments in the field of omics technologies like DNA microarray, MALDI, SILAC has further provided newer insight into the underlying mechanisms for the changes in gene copy number either by gene deletion, amplification or gene rearrangements.

Emergence and spreading of drug resistance can dramatically jeopardize the VL control program relying on chemotherapy, as shown in malaria. It is thought that inadequate treatment, either regimen or treatment duration, is the main cause for failure of treatment and resulting in emergence of drug resistance [102]. Nothing is known about the dynamics of drug resistant *Leishmania* populations in the presence or absence of drug pressure. Therefore, monitoring of drug efficacy and early reporting are essential to bring corrective actions in drug policy. This requires tools, a standardized way to use them and a structure to implement them: in the mid-term, a network of sentinel sites could be established by the VL elimination program, like it was done in East Africa for malaria [103].

## What are the possible solutions?

Although considerable scientific progress has been made over the past decade in the broad domain of leishmaniasis, including the genome sequencing of various pathogens causing different form of leishmaniasis, these have not had any impact so far on the quality of clinical care for VL in the field due to very limited number of available anti-leishmanial drugs. It is very important to safeguard the effectiveness of these drugs in order to cure patients and to sustain the VL control. For this, the uninterrupted supply of quality drugs, the promotion of treatment adherence and the monitoring of treatment effectiveness as well as drug resistance will be pivotal. One of the major hurdles for identifying VL patients who are unlikely to respond adequately to chemotherapy, has been a limited understanding of the precise immune mechanisms required for controlling parasite growth, particularly the immune mechanisms that are generated following drug treatment. Anti-leishmanial drugs as monotherapy are high risk of emergence of resistant parasites [16], therefore multidrug therapy needs to be recommended. Another important problem is disease relapse following drug treatment, and at present there are no good prognostic markers to identify individuals that might fail drug treatment. Because the knowledge on mechanisms of emergence of drug resistance, its dynamics and the impact of the introduction of new drugs is poor, and validated methods to monitor treatment of effectiveness under routine conditions do not exist, it is therefore very urgent to develop new tools to allow monitoring treatment effectiveness and drug resistance in order to support the drug policy of the VL elimination program. Furthermore, treatment outcome (the end of treatment) is not definite and patients need to be followed up 6 months after treatment to assess cure. This makes monitoring treatment effectiveness in routine conditions difficult, as patients may not come back for the requested visit 6 months post-treatment. Hence there is a need to compare existing and develop new approaches to monitor treatment effectiveness at the program level. Several possibilities exist: to work with proxy indicators, to develop a method adapted from the retrospective cohort analysis used in TB programs, to give incentives as transport allowances to patients etc.

Importantly, patients with PKDL represent an important but largely neglected reservoir of infection that perpetuates anthroponotic *Leishmania donovani* disease in India, and focal VL outbreaks have been linked to an index case of PKDL [104]. Treatment of PKDL in India is widely regarded as unsatisfactory, and the low incidence of PKDL in India makes any prospective clinical study challenging. Lack of animal models for PKDL is another challenge for laboratory testing of new drugs. Therefore, more research on an intervention that can reduce the risk of developing PKDL; and characterization of parasite strain are needed to resolve the mystery of this disease.

The use of chemotherapy alone as control tool is limited by the fact that only sick people will be treated. There are asymptomatic carriers of the parasite (estimated 6 times more than VL patients) [105–107], and in the absence of chemoprophylaxis of leishmaniasis, these will obviously not be exposed to the drug [3]. Exact role of asymptomatic infection in disease transmission is unknown, but control programs should take them into consideration, hence role of asymptomatic should be quickly elucidated in context to VL transmission.

Last but not the least, not only VL treatment programs should be maintained and improved, but research should also be taken into consideration those parasite reservoirs in populations in order to reduce the risk of transmission. Programs based on management of vector control should be continued as a critical part of treatment strategy. Research on development of safe and effective vaccine have to be promoted that could make a significant impact on the re-emergence of VL cases and sustain the transmission level in endemic areas.

## Conclusion

In the absence of effective vaccine and vector control measures, control of VL and PKDL almost exclusively depends on chemotherapy. The available drugs are costly and may require hospitalization that needs monitoring which cause substantial loss of income for affected families. Emergence of drug resistance further complicates the treatment of disease. Multidrug regimens for VL hold much promise and, experiences with single dose L-AmB are excellent and this will have obvious benefits to the patients who will not require hospital care and loss of wages. However, more studies are required on treatment of PKDL and HIV-VL co-infections as they serve as silent reservoir in endemic areas, and as such will jeopardize the sustainability of VL elimination in the ISC. We have now entered in the VL elimination year, and it is best time to repeat the experience of smallpox and polio eradication in order to open a whole new public health era for next generations.

## Additional file

Additional file 1:
**Multilingual abstracts in the six official working languages of the United Nations.** (PDF 277 kb)

## Abbreviations

AmB: Amphotericin B deoxycholate
CD: cluster of differentiation
ERK: extra cellular signal regulated kinases
i.v: intravenously
IL-10: interleukin-10
IM: intramuscularly
ISC: Indian subcontinent
L-AmB: Liposomal Amphotericin B
MAPK: mitogen activated protein kinases
M-CSF: macrophase colony stimulating factor
MDR: multi drug resistance
NO: nitric oxide
PKDL: post kala-azar dermal leishmaniasis
R&D: research and development
ROS: reactive oxygen species
SSG: sodium stibogluconate
TCA: tri cyclic acid cycle
TNF: Tumor necrosis factor
VL: visceral leishmaniasis
