# Supplementary material for: Current challenges in treatment options for visceral leishmaniasis in India: a public health perspective
Source: Infect Dis Poverty. 2016 Mar 8;5:19. doi: 10.1186/s40249-016-0112-2 (PMC4782357; doi:10.1186/s40249-016-0112-2)
Supplement: Additional file 1: — Multilingual abstracts in the six official working languages of the United Nations. (PDF 277 kb) [file 40249_2016_112_MOESM1_ESM.pdf]

Translation of the abstract into the six official working languages of the United Nations

تحديات الخيارات العلاجية الحالية لداء الليشمانيات الحشوي في الهند: منظور الصحة العامة

أوم براكاش وبهوانا سينج، جايا تشاكرافارتي وشيامساندر.

الملخص

يُعد داء الليشمانيات الحشوى (VL) من الأدواء المدارية المهُملة من حيث تطوير العقاقير الجديدة، ويرجع ذلك إلى افتقار المردود المادي. لاتزال حملة القضاء على المرض في الهند جارية منذ عام 2005، وتهدف إلى تقليل نسبة الإصابة بمرض (VL) إلى أقل من شخص واحد بين عشرة آلاف شخصًا وذلك على مستوى المناطق الفرعية. من الوسائل التي تبنتها الحكومة كخطوة للقضاء على المرض كانت التشخيص المبكر والعلاج الكامل. أحرز علاج داء الليشمانيات الحشوي (VL) تقدمًا ملحوظًا على مدى السنوات القليلة الماضية، فقد أُثبت أن جرعة واحدة من دهون الأمفوتيريسين B الشحمي (liposomal Amphotericin-B) بالإضافة إلى العلاجات التي تعتمد على أدوية عدة يعتبرا الخيار العلاجي الأمثل في شبه القارة الهندية. ولكن تظل هناك تحديات عدة للتغلب عليها. ومن أمثلة تلك التحديات: توافر العقاقير وتكلفة العلاج (العقاقير والإيداع بالمشفى وزيادة المقاومة ضد الطفيليات. لذلك، فمن الأفضل التركيز على تنفيذ الأبحاث بسرعة؛ لتحديد أفضل السبل لتقديم التدخلات القائمة، بما في ذلك التشخيص والعلاجات المتاحة لداء (VL). من الضروري أن تتوفر الخيارات العلاجية الجديدة، ولا تنحصر فقط على المناطق الموبوءة حتى تروج لنسبة الشفاء وإنقاذ الأرواح.

Translated from English version into Arabic by Yousra Fakhrey, through

当前印度内脏利什曼原虫病治疗面临的挑战：公共卫生观点

Om Prakash Singh, Bhawana Singh, Jaya Chakravarty and Shyam Sundar

摘要

内脏利什曼原虫病（VL）是被忽视的热带病之一，特别是该类新药开发经济回报较低。2005年印度开始实施VL消除行动，其目的是将乡镇水平VL发病率降低至万分之一以下。早期诊断和全程治疗是政府采取消除行动计划的重要措施之一。近年来，VL治疗方面已经取得了实质性的进展，单剂两性霉素B脂质体和联合疗法是印度次大陆首选的治疗方案。然而，仍需克服药物可获得性、治疗成本（药物和住院）以及逐渐增强的原虫耐药性等挑战。因此，更好地着重于应用研究是当前的迫切需求，以便确定如何最优化实施现有的包括VL诊断和治疗的干预措施。新的治疗方案切实易获得是重点，而不是简单的在流行地区可获得，用以促进康复和拯救生命。该综述覆盖了当前VL流行区治疗方案的新进展和挑战，探讨了提高临床预后的可能策略。

Translated from English version into Chinese by Chen Tianmu, edited by Yang Pin, through

Les difficultés actuelles dans le choix d'un traitement contre la leishmaniose viscérale en Inde : le point de vue de la santé publique

Om Prakash Singh, Bhawana Singh, Jaya Chakravarty and Shyam Sundar

Résumé

La leishmaniose viscérale est une maladie tropicale négligée, en particulier sur le plan du développement de nouveaux médicaments, en raison de sa faible rentabilité. Une campagne d'élimination est en cours en Inde depuis 2005 ; son but est de réduire l'incidence de la leishmaniose viscérale à moins de 1 personne pour 10 000 au niveau des sous-districts. Le gouvernement compte, entre autres, sur un diagnostic précoce suivi d'un traitement complet pour éliminer la maladie. Des progrès considérables ont été faits ces dernières années dans le traitement de la leishmaniose viscérale. L'amphotéricine B liposomique en dose unique et des multithérapies médicamenteuses constituent les meilleures options envisagées dans le sous-continent indien. De nombreux obstacles doivent cependant encore être surmontés : disponibilité des médicaments, coût du traitement (médicaments et hospitalisation), résistance croissante du parasite. Des travaux de recherche sur le déploiement sont donc nécessaires d'urgence afin de déterminer le meilleur moyen de déployer les interventions existantes, notamment les moyens de diagnostic et de traitement disponibles contre la leishmaniose viscérale. Afin de favoriser la guérison et sauver des vies, il est essentiel que les nouvelles options de traitement soient véritablement accessibles, et pas uniquement dans les zones d'endémies. Notre revue couvre les récents progrès et les difficultés dans les options actuelles de traitement de la leishmaniose viscérale dans les régions d'endémie et discute des stratégies possibles pour améliorer les résultats cliniques.

Translated from English version into French by Suzanne Assenat, through

TRANSLATORS
WITHOUT BORDERS

Существующие проблемы лечения висцерального лейшманиоза в Индии: перспективы развития государственной системы здравоохранения

Ом Пракаш Синх, Бхавана Синх, Джайя Чакраварты и Шъям Сундар

Краткий обзор

Висцеральный лейшманиоз – это тропическое заболевание, лечение которого явно недооценивают по причине его нерентабельности, особенно что касается разработки новых препаратов. Начиная с 2005 года в Индии проходит кампания по борьбе с данным заболеванием, цель которой состоит в том, чтобы сократить уровень заболеваемости висцеральным лейшманиозом до 1 человека на 10000 жителей на подрайонном уровне. Ранняя диагностика в сочетании с последующим комплексным лечением – это один из компонентов, который используется правительством для борьбы с болезнью. В последние годы удалось достичь значительного успеха в области лечения висцерального лейшманиоза: введение одной дозы липосомного амфотерицина Б и одновременное применение нескольких лекарственных препаратов являются лучшими способами, используемыми на индийском субконтиненте. Однако, предстоит решить

еще много проблем. К ним относятся доступность лекарственных средств, стоимость лечения (медикаменты и госпитализация) и растущая резистентность паразитов. Таким образом, необходимо как можно скорее направить все силы на исследование проблемы с тем, чтобы определить наилучшие способы реализации существующих методов, включая доступную диагностику и способы лечения висцерального лейшманиоза. Важно, чтобы новые способы лечения стали доступными повсеместно, а не только в зонах распространения заболевания – только так можно стимулировать процессы лечения и спасения жизней. Настоящий обзор рассказывает о последних достижениях и проблемах в области способов лечения висцерального лейшманиоза в зоне распространения заболевания и инициирует обсуждение возможных стратегий улучшения клинических результатов.

Translated from English version into Russian by Irina Zayonchkovskaya, through

Retos que presentan actualmente las opciones de tratamiento para la leishmaniasis visceral en India desde la perspectiva de la sanidad pública

Om Prakash Singh, Bhawana Singh, Jaya Chakravarty y Shyam Sundar

Resumen

La leishmaniasis viral (LV) es una enfermedad tropical olvidada, especialmente en lo que se refiere al desarrollo de nuevos fármacos, que no reportarán beneficios económicos. En India se está llevando a cabo una campaña de erradicación desde 2005, con el fin de reducir la incidencia de la LV por debajo del 1 por 10000 en los subdistritos. Un diagnóstico precoz, seguido de un tratamiento completo, son algunas de las medidas adoptadas por el gobierno para erradicar la enfermedad. En los últimos años se han realizado importantes avances en el tratamiento de la LV, y actualmente el tratamiento de dosis única con Anfotericina B liposomal, así como los tratamientos multimedicamentosos, constituyen las mejores opciones en el subcontinente indio. Sin embargo, aún quedan muchos retos a los que hacer frente, entre los que se incluyen la disponibilidad de los fármacos, el coste del tratamiento (medicación y hospitalización) y la creciente resistencia de los parásitos. Por ello, es necesario fomentar urgentemente el desarrollo de una línea de investigación aplicada que permita determinar la mejor manera de poner en práctica las medidas existentes, incluyendo los diagnósticos y tratamientos disponibles contra la LV. Es imprescindible que las nuevas opciones de tratamiento sean realmente accesibles en las zonas endémicas, en lugar de estar simplemente disponibles, para que puedan curar a los enfermos y salvar vidas. Este análisis cubre los recientes avances y los desafíos que presentan actualmente las opciones de tratamiento para la LV en las zonas de enfermedades endémicas, y aborda posibles estrategias para mejorar los resultados clínicos.

Translated from English version into Spanish by Yaiza Jurado, through
